# 5.M. Workshop: The need to account for vaccine-specificities in the implementation of the EU Regulation on HTA

**DOI:** 10.1093/eurpub/ckac129.326

**Published:** 2022-10-25

**Authors:** Organised by: EUPHA-HTA, Vaccines Europe, HTAi, Chair persons: Chiara de Waure (EUPHA-HTA), Elena Petelos (EUPHA-HTA)

## Abstract

In January 2022, the EU Regulation on Health Technology Assessment (HTAR) entered into force. The HTAR provides for the centralized conduct of EU-wide joint clinical assessment (JCA) for oncology treatments (2025 onwards), and for other medicinal products incl. vaccines (2030 onwards). HTA is a multidisciplinary process aiming to disentangle the value of a health technology in comparison to other. It has been used, to different extent across EU countries, to inform vaccine price and reimbursement decisions. Nevertheless, existing assessment frameworks utilized by HTA bodies, incl. National Immunization Technical Advisory Groups (NITAGs), as well as the relative effectiveness assessment (REA) framework developed by the European Network for Health Technology Assessment (EUnetHTA), do not fully account for the specificities of vaccines. In fact, to account for the broad value of vaccines several aspects ought to be considered, including societal benefits. Attention has been paid to these topics for years by the public health community, and in particular EUPHA-HTA, and also Vaccines Europe, a specialized vaccines group within the European Federation of Pharmaceutical Industries and Associations (EFPIA), has recently released a position statement on the need to tackle vaccine specificities in future JCA. Furthermore, as the COVID-19 pandemic has highlighted, such aspects are critical for joint procurement efforts to combat the current and future pandemics. The objective of this workshop is to address the current and future role of HTA in vaccine regulation across EU countries, inform on key specificities of vaccines in respect to JCA and HTA as a whole and discuss opportunities to develop vaccine-specific methodological guidelines within the HTAR. Following an introduction, the following topics will be addressed:

• Competent bodies and HTA impact on vaccine decision-making process, incl. a discussion of implementation challenges and potential impacts of the HTAR.

• JCA specificities in respect to vaccines and the importance of addressing the whole value of vaccines and vaccinations.

• The proposal of Vaccines Europe for guiding principles to account for vaccines specificities in JCA built on the outcomes of an ‘across-the-industry’ project incl. also non-industry experts.

The presentations will be followed by a panel discussion to further explore the themes, consider next steps and address questions from the audience.

**Key messages:**

• The new EU HTAR is paving the way for JCAs of several medicinal products, incl. vaccines necessitating expert guidance in terms of determining their whole value.

• Vaccines specificities should be duly considered in JCAs to inform national and EU-wide decision-making for vaccination programmes, reimbursement, and pricing.

